# 

**Published:** 1909-11-15

**Authors:** 


CONTENTS

LIBRARY
Indexed
S. G. O.

Event and Comment - - - - - - - 551

Syphilis, with Special Reference to Recent Experimental Work,

*By Henry Rockwell Varney, M.D.* 555

Cresyl Formothymol in the Treatment of Caries of the Third and Fourth Degree, - - - - *By Dr. V. E. Miegeville* 586

Indents - - - - - - - - - - - 591

Announcements - - - - - - - - - 603

